# Clinical and Functional Characterization of a Novel Mutation in Lamin A/C Gene in a Multigenerational Family with Arrhythmogenic Cardiac Laminopathy

**DOI:** 10.1371/journal.pone.0121723

**Published:** 2015-04-02

**Authors:** Cinzia Forleo, Monica Carmosino, Nicoletta Resta, Alessandra Rampazzo, Rosanna Valecce, Sandro Sorrentino, Massimo Iacoviello, Francesco Pisani, Giuseppe Procino, Andrea Gerbino, Arnaldo Scardapane, Cristiano Simone, Martina Calore, Silvia Torretta, Maria Svelto, Stefano Favale

**Affiliations:** 1 Cardiology Unit, Department of Emergency and Organ Transplantation, University of Bari, Bari, Italy; 2 Department of Biosciences, Biotechnology and Biopharmaceutics, University of Bari, Bari, Italy; 3 Section of Medical Genetics, Department of Biomedical Sciences and Human Oncology, University of Bari, Bari, Italy; 4 Department of Biology, University of Padua, Padua, Italy; 5 Section of Radiology, Interdisciplinary Department of Medicine, University of Bari, Bari, Italy; Tokyo Medical and Dental University, JAPAN

## Abstract

Mutations in the lamin A/C gene (*LMNA*) were associated with dilated cardiomyopathy (DCM) and, recently, were related to severe forms of arrhythmogenic right ventricular cardiomyopathy (ARVC). Both genetic and phenotypic overlap between DCM and ARVC was observed; molecular pathomechanisms leading to the cardiac phenotypes caused by *LMNA* mutations are not yet fully elucidated. This study involved a large Italian family, spanning 4 generations, with arrhythmogenic cardiomyopathy of different phenotypes, including ARVC, DCM, system conduction defects, ventricular arrhythmias, and sudden cardiac death. Mutation screening of *LMNA* and ARVC-related genes *PKP2*, *DSP*, *DSG2*, *DSC2*, *JUP*, and *CTNNA3* was performed. We identified a novel heterozygous mutation (c.418_438dup) in *LMNA* gene exon 2, occurring in a highly conserved protein domain across several species. This newly identified variant was not found in 250 ethnically-matched control subjects. Genotype-phenotype correlation studies suggested a co-segregation of the *LMNA* mutation with the disease phenotype and an incomplete and age-related penetrance. Based on clinical, pedigree, and molecular genetic data, this mutation was considered likely disease-causing. To clarify its potential pathophysiologic impact, functional characterization of this *LMNA* mutant was performed in cultured cardiomyocytes expressing EGFP-tagged wild-type and mutated LMNA constructs, and indicated an increased nuclear envelope fragility, leading to stress-induced apoptosis as the main pathogenetic mechanism. This study further expands the role of the *LMNA* gene in the pathogenesis of cardiac laminopathies, suggesting that *LMNA* should be included in mutation screening of patients with suspected arrhythmogenic cardiomyopathy, particularly when they have ECG evidence for conduction defects. The combination of clinical, genetic, and functional data contribute insights into the pathogenesis of this form of life-threatening arrhythmogenic cardiac laminopathy.

## Introduction

Lamins A and C, encoded by the lamin A/C gene (*LMNA*), are major structural components of the nuclear lamina, a protein meshwork supporting the inner nuclear membrane [1]. In addition to sustaining the structural integrity and mechanical stability of the nuclear envelope, lamins are involved in multiple cellular processes, such as chromatin organization, DNA replication, gene regulation, and nucleo-cytoskeletal coupling [2]. *LMNA* gene mutations are implicated in a wide spectrum of laminopathies, inherited diseases characterized by phenotypic heterogeneity, including cardiac and skeletal myopathies, lipodystrophy, peripheral neuropathy, and premature aging syndromes [1, 3].

The cardiac phenotype of laminopathies is characterized by conduction system disorders (CD), arrhythmias, and dilated cardiomyopathy (DCM) [4]. Many *LMNA* mutation carriers have a poor prognosis [5], due to a high rate of major cardiac events, such as sudden cardiac death (SCD), life-threatening ventricular arrhythmias, extreme bradycardia due to high-degree atrioventricular block, and progression to end-stage heart failure [4]. In addition to *LMNA* DCM-CD, some atypical forms of *LMNA*-related cardiac diseases were reported [6, 7]. Recently, severe forms of arrhythmogenic right ventricular cardiomyopathy (ARVC) have been linked to lamin A/C gene mutations [8], and both genetic and phenotypic overlap between DCM and ARVC was observed [8–11]. Although the role of lamins in cell functions has been widely investigated, the pathophysiological mechanisms leading to cardiac phenotypes caused by *LMNA* mutations are not yet fully understood [1–3].

In this study, we detected a novel *LMNA* gene mutation in a large family with arrhythmogenic cardiomyopathy of different phenotypes, including ARVC, DCM, conduction disturbances, arrhythmias, and sudden cardiac death (SCD). We investigated the involvement of the *LMNA* gene in the pathogenesis of this arrhythmogenic, familial cardiac laminopathy and functionally characterized the newly-identified *LMNA* mutant.

## Materials and Methods

### Ethics Statement

All participants provided written informed consent. The Ethics Committee of University Hospital Consortium, Policlinico of Bari, Italy approved the study. This study conforms to the principles outlined in the Declaration of Helsinki (World Medical Association Declaration of Helsinki).

### Clinical Evaluation

The study involved 20 individuals from 4 generations of a large Italian family, referred to our Unit dedicated to Cardiomyopathies. The 54-year-old male index patient was diagnosed in 2001 with ARVC, based on the original Task Force criteria (TFC) of 1994 [12].

Family cascade screening was performed [13]. All subjects underwent clinical workup, including medical history, physical examination, measurement of serum creatine phosphokinase, 12-lead electrocardiogram (ECG), transthoracic echocardiography, 24-hour ECG recording, and exercise testing. When appropriate, coronary angiography was performed. Subjects without contraindication to magnetic resonance imaging, such as pacemaker, defibrillator, or severe claustrophobia underwent cardiac magnetic resonance (CMR) (for details, see S1 Methods).

### Genetic Analysis and Mutation Detection

Genomic DNA was obtained from peripheral blood samples using the Wizard Genomic DNA Purification kit (Promega Corporation, Madison, Wisconsin, USA), as recommended by the manufacturer. Mutation screening of plakophilin-2 *(PKP2*), desmoplakin (*DSP*), desmoglein-2 (*DSG2*), desmocollin-2 (*DSC2*), plakoglobin (*JUP*), and αT-catenin (*CTNNA3*) genes was performed as previously reported [14, 15]. All coding exons of *LMNA* gene were amplified by PCR and analyzed by High Resolution Melting, as reported in G. Millat et al [16]. The PCR products of exon 2 and 10 were analyzed by direct sequencing on a 310 ABI sequencer. Numbering of the *LMNA* nucleotides refers to GenBank accession number NM_170707.2. A control group of 250 healthy and unrelated Italian subjects (500 alleles) was used to exclude the possibility that any identified variation could be due to DNA polymorphism. All controls were unrelated healthy volunteers matched to the index patient by ancestry from the general Italian population. Moreover, all identified variants were systematically searched for in dbSNP (http://www.ncbi.nlm.nih.gov/projects/SNP/), in the 1000 Genomes Project database (http://www.1000genomes.org), or in the Exome Variant Server (http://evs.gs.washington.edu/EVS/).

### Functional Studies in HL-1 Cardiomyocytes

The functional characterization of this mutated lamin A protein (LMNA) was performed in cultured HL-1 cardiomyocytes expressing EGFP-N-terminal-tagged wild-type (WT) and mutated LMNA. Live-imaging experiments were carried out in a BioStation IM device (Nikon). The acquisition timing was set to every 5 minutes for 16–32 hours, and up to 10 cell fields were captured at each time point. Hyperosmotic stress was induced by incubating HL-1 cells in culture medium with 300 mM mannitol for 2h. Hypoxic stress was performed in a Hypoxia Modular Incubator Chamber (Billups-Rothenberg Inc) and a flow rate of 4 liters/minute of 100% N_2_ was applied for 15 min. Cells, in the hypoxia chamber saturated with N_2_, were placed at 37°C for 8h. Oxidative stress was induced by incubating HL-1 cells in culture medium plus 300 μM H_2_O_2_ for 4 h.

For further details, see S1 Methods.

### Statistical Analysis

Continuous variables were expressed as mean values ± standard deviation, and frequencies as the number and percentage of patients. Between-group comparisons were made using the non-parametric Wilcoxon rank-sum test. Frequencies were compared using the Fisher’s exact test. The analyses were performed using STATA software, version 12 (StataCorp, College Station, TX, USA). A P-value of <0.05 was considered statistically significant.

## Results

### Genetic Analysis

**Fig 1:**
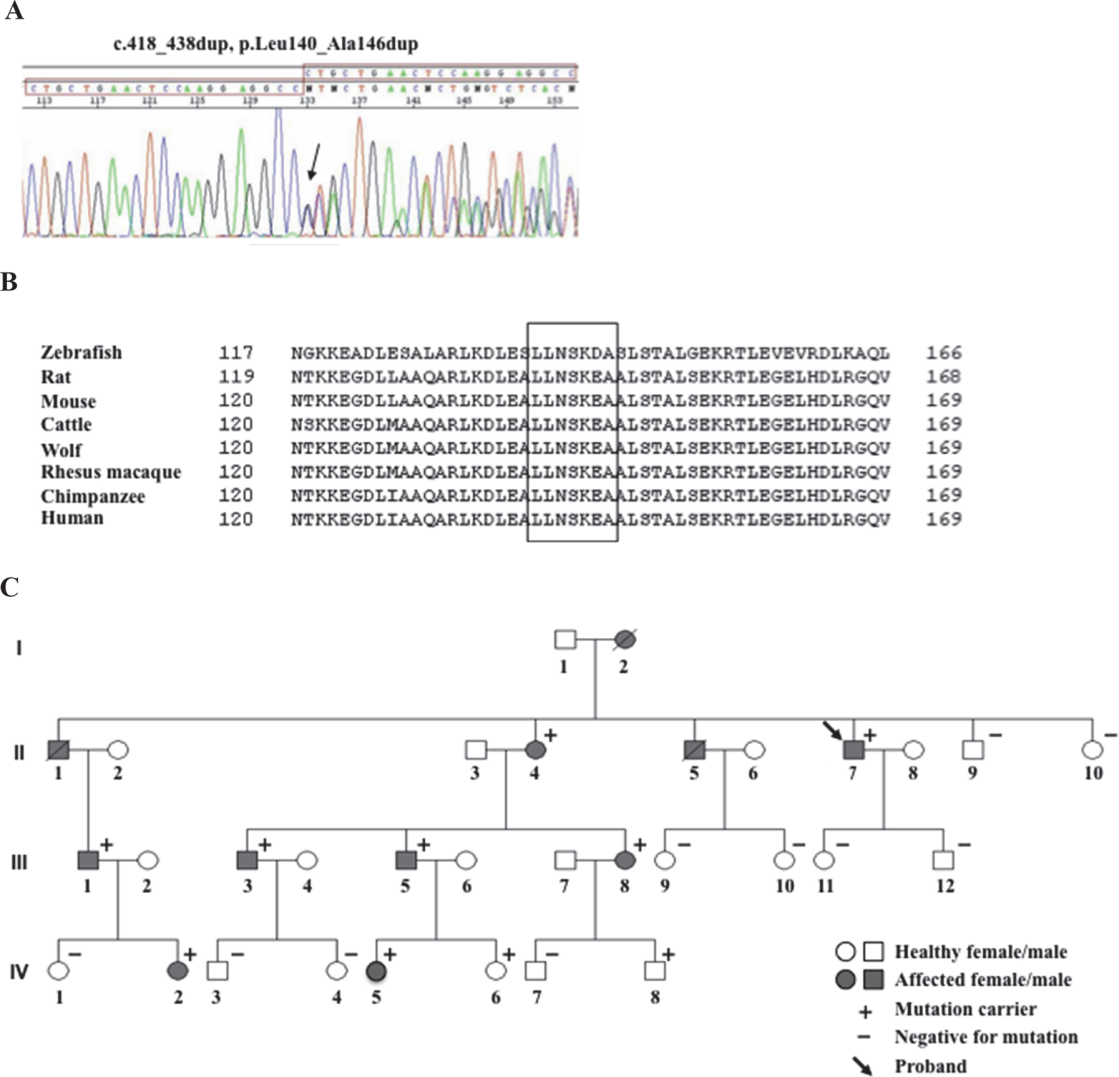
Family pedigree and mutation identification. (A) Electropherogram of the *LMNA* gene variant. (B) The 7 duplicated amino acids (LLNSKEA) are highly conserved among *LMNA* gene homologs in vertebrates. (C) Family pedigree of the index patient with the novel *LMNA* gene mutation.

We identified in the index patient the novel heterozygous lamin A/C variant, c.418_438dup, p.Leu140_Ala146dup, consisting of a duplication of 21 nucleotides (CTGCTGAACTCCAAGGAGGCC) in exon 2 of the *LMNA* gene (Fig. 1A), located in the coil 1B of the central α-helical rod domain of the lamin A/C protein. The *LMNA* variant is predicted to result in the duplication of seven amino acids (LLNSKEA), from position 140 to position 146, in the lamin A/C protein, without a frame shift in the open reading frame and affects a highly conserved amino acid region across several species (Fig. 1B), suggesting a possible pathogenetic role.

This *LMNA* variant was subsequently detected in 10 of 20 family members who underwent family cascade genetic screening (Fig. 1C). The index patient (subject II-7 in Fig. 1C) was also screened for mutations in the ARVC-related genes *PKP2*, *DSP*, *DSG2*, *DSC2*, *JUP*, and *CTNNA3* without positive findings.

The novel *LMNA* variant was not present in 250 healthy control individuals nor found in the above- listed GenBank databases.

### Clinical Findings

**Fig 2:**
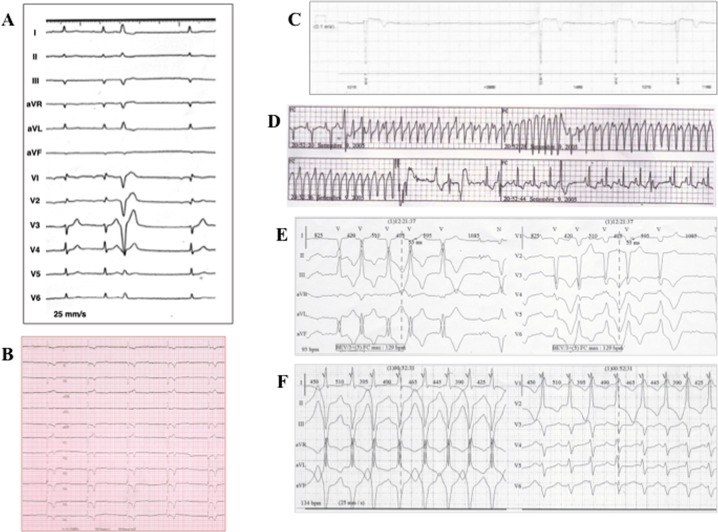
Clinical characteristics of index patient and *LMNA* mutation-positive family members. Index patient’s electrocardiogram (ECG) showing, at clinical presentation, (A) sinus rhythm, first-degree AV block, and PVC of LBBB morphology and, 13 years later, (B) complete AV block. (C) Asystole documented on implantable loop recorder memory (subject III-8). (D) Sustained VT detected on telemetry monitoring, effectively terminated by internal ICD shock (subject III-1). (E) Episodes of non-sustained VT with LBBB morphology and inferior axis (subject III-5) on 12-lead Holter monitoring. (E) Episodes of non-sustained VT with RBBB morphology and superior axis (subject IV-2) on 12-lead Holter monitoring.

Pedigree structure (Fig. 1C) and clinical characteristics of all evaluated subjects (Table 1) are presented. ECG, Holter, and cardiac structural abnormalities of family members carrying the *LMNA* mutated variant are summarized in Table 2. Pedigree was consistent with autosomal dominant transmission (Fig. 1C). The index patient (II-7) in 2001 presented with palpitation. In the ECG, first-degree atrioventricular (AV) block and premature ventricular complexes (PVCs) of left bundle-branch block (LBBB) morphology were detected (Fig. 2A). Frequent (up to 35000 per day) multifocal PVCs and runs of non-sustained ventricular tachycardia (NSVT) were recorded using 24-hour Holter monitoring. An echocardiogram showed normal size and preserved global function of both ventricles. Coronary angiography was normal, and right ventricle (RV) angiography showed dyskinetic areas with bulging at the RV free wall. On the basis of clinical and instrumental features fulfilling the presence of 1 major plus 2 minor criteria, the index patient was diagnosed with ARVC, based on the original TFC [12], and received a prophylactic implantable cardioverter-defibrillator (ICD). He had a positive family history for DCM and SCD; his mother (I-2) died suddenly at rest at the age of 39 years and his brother (II-1) died from SCD at the age of 43 years while playing soccer. No autopsy data were available. Another brother (II-5), suffering from DCM, underwent cardiac transplantation at the age of 48 years, and died 14 years later due to refractory heart failure.

**Table 1:** Clinical characteristics of family members according to *LMNA* mutation carrier status.

	All subjects (n = 20)	***LMNA*** mutation-positive subjects (n = 10)	***LMNA*** mutation-negative subjects (n = 10)	***P*** value
Age (years)	36±16	35±17	37±16	0.910
Male gender, n (%)	9 (45)	5 (50)	4 (40)	1.000
SBP (mm Hg)	116±11	117±12	116±9	0.969
DBP (mm Hg)	75±10	76±7	75±12	0.535
BMI (Kg/m^2^)	23.8±3.9	25±4.3	22.6±3.2	0.199
Heart rate (bpm)	65±11	60±6	70±13	**0.028**
PR interval (msec)	196±72	231±88	161±23	0.109
QRS duration (msec)	86±8	87±10	86±6	0.866
QTc interval (msec)	406±19	402±13	410±23	0.540
AF, n (%)	2 (10)	2 (20)	0 (0)	0.474
AV block, n (%)	7 (35)	7 (70)	0 (0)	**0.003**
PVCs >500/24h, n (%)	7 (35)	7 (70)	0 (0)	**0.003**
PVCs >200/24h, n (%)	9 (45)	9 (90)	0 (0)	**0.001**
NSVT, n (%)	6 (30)	6 (60)	0 (0)	**0.011**
SVT, n (%)	2 (10)	2 (20)	0 (0)	0.474
LVEF (%)	60±7	56±8	64±4	**0.012**
LVEDD (mm)	44.4±4.7	46.2±5.6	42.6±3.0	0.095
RVOT (mm) by echo*	25.3±2.7	25.4±3.0	25.1±2.6	0.680
RVEF (%) by CMRI†	45.6±4.4	42.9±4.8	47.9±2.3	**0.020**
LGE on CMRI, n (%)	4 (27)	4 (57)	0 (0)	**0.026**

Mean values ± standard deviation or absolute frequencies and percentage of patients.

AF = atrial fibrillation; AVB = atrioventricular block; BMI = body mass index; CMRI = cardiac magnetic resonance imaging; DBP = diastolic blood pressure; Echo = echocardiography; LGE = late gadolinium enhancement; LVEDD = left ventricular end-diastolic diameter; LVEF = left ventricular ejection fraction; NSVT = non-sustained ventricular tachycardia; PVCs = premature ventricular complexes; RVEF = right ventricular ejection fraction; RVOT = right ventricular outflow tract; SBP = systolic blood pressure; SVT = sustained ventricular tachycardia.

*RVOT value by echocardiography was available in 19 of 20 patients, 9 of whom were *LMNA* mutation-positive subjects.

†CMRI was performed in 15 subjects, 7 of whom were *LMNA* mutation carriers.

**Table 2:** Clinical characteristics of *LMNA* mutation-positive family members.

**Sub**	Gen	Age*	Symptoms	PR interval (msec)	Conduction system defects	Ventricular arrhythmias**	RVOT by Echo (mm)	RVEF by CMR (%)	LVEF (%)	LVEDD (mm)	LGE-CMR	TFC***	Com
II-7	M	54	Palpitation	230	I°-II°-III° AVB; paroxismal AF	PVCs 35000/24h; NSVT of LBBB morphology with superior axis	29	np	55	47	np	1/2	ICD
II-4	F	58	Palpitation	380	SB; I°-II° AVB; permanent AF	PVCs 912/24h	26	np	60	47	np	0/2	PM
III-1	M	35	Palpitation and dyspnea, followed by syncope on effort	200	II°AVB	PVCs 5000/24h; NSVT; SVT	np	np	40	59	np	0/2	ICD;HT
III-3	M	47	Palpitation	340	SB; I°-II° AVB; poor R-wave progression	PVCs 651/24h; NSVT	28	41	56	45	**+**	0/1	ICD
III-5	M	45	Palpitation	270	I°-II° AVB; poor R-wave progression	PVCs 1239/24h; NSVT of LBBB morphology with inferior axis; NSVT of RBBB morphology with superior or inferior axis	27	38	48	48	**+**	1/1	ICD
III-8	F	42	Asymptomatic	300	SB; I° AVB; poor R-wave progression; 3.5 sec asymptomatic asystole	PVCs 380/24h; NSVT of RBBB morphology with superior axis	25	45,5	63	44	**+**	0/1	ILR
IV-2	F	23	Syncope	160	poor R-wave progression	PVCs 1074/24h; NSVT of LBBB morphology with inferior axis; NSVT of RBBB morphology with superior axis; SVT	24	38	55	49	**+**	0/1	ICD
IV-5	F	20	Asymptomatic	140		PVCs 2237/24h	20	41	63	41	-	0/1	FU
IV-6	F	14	Asymptomatic	140		PVCs 234/24h	22	51	65	38	-	0/1	FU
IV-8	M	12	Asymptomatic	150	SB; rare nocturnal episodes of I° and Mobitz 1 II° AVB	PVCs 5/24h	28	46	59	44	-	0/0	FU

AF = atrial fibrillation; ARVC = arrhythmogenic right ventricular cardiomyopathy; AVB = atrioventricular block; CMR = cardiac magnetic resonance imaging; Com = comments; Echo = echocardiography; EF = ejection fraction; F = female; FU = Follow-up; Gen = Gender; HT = heart transplantation; ICD = implantable cardioverter-defibrillator; ILR = implantable loop recorder; LBBB = left bundle-branch block; LGE = late gadolinium enhancement; LV = left ventricle; LVEDD = left ventricular end-diastolic diameter; M = male; m = minor; Mj = major; np = not performed; NSVT = non-sustained ventricular tachycardia; PM = pacemaker; PVCs = premature ventricular complexes; RBBB = right bundle-branch block; RV = right ventricle; RVOT = right ventricular outflow tract; SB = sinus bradycardia; Sub = Subject; SVT = sustained ventricular tachycardia; TFC = Task Force criteria.

*at diagnosis/clinical presentation (years);

**Morphology of NSVT was determinable in 4 of 6 *LMNA* mutation-positive family members having NSVT;

***for ARVC diagnosis Mj/m.

Among the 10 *LMNA* mutated variant carriers, subject II-4 in 2002, at the age of 58 years, received a pacemaker due to atrial fibrillation (AF) with a slow ventricular response alternating with sinus bradycardia. Subject III-1 developed DCM. Subject III-5, who fulfilled modified TFC for borderline ARVC diagnosis [17], showed left ventricle (LV) systolic dysfunction without dilatation (Table 2). The distribution of major and minor criteria, according to the modified TFC [17], is reported in Table 2.

Abnormal ECG findings were present only in family members carrying the mutated *LMNA* variant, seven (70%) of whom had, at clinical presentation or went on to develop, conduction disturbances (sinus bradycardia and/or first-, second-, or third-degree AV block) (Table 2; Fig. 2A, B, and C); 2 subjects developed AF.

Ventricular arrhythmias were documented in 9 of 10 (90%) *LMNA* mutation-positive subjects, and ranged from sustained VT (n = 2), detected on telemetry (Fig. 2D) or ICD memory, to monomorphic NSVT with LBBB (n = 3) (Fig. 2E) and/or right bundle-branch block (RBBB) morphology (n = 3) (Fig. 2F), and polymorphic PVCs (n = 9) (Table 2), recorded on Holter monitoring. The total number of PVCs ranged from 234 to 35000/24 h. Considering the modified criteria for familial ARVC [17], 9 (90%) individuals had >200 PVCs in 24 hours, and 7 (70%) had >500 PVCs in 24 hours (Tables 1 and 2). One of the 2 patients with sustained VT, and 5 of 6 (83%) patients with NSVT, had concomitant AV block (Table 2).

Echocardiographic findings revealed LV involvement in 2 *LMNA* mutation carriers (Table 2). Patient III-1, who had LV borderline systolic function (LVEF = 55%) at clinical presentation at the age of 35, developed global LV hypokinesia (LVEF = 40%) and enlargement 6 years later (Table 2). Individual III-5, who had normal LV systolic function (LVEF = 60%) on echocardiography during familial screening at the age of 36, presented LV systolic dysfunction (LVEF = 48%) without LV enlargement 9 years later (Table 2). Taken together, mean LVEF values were relatively preserved (56% ± 8%) in *LMNA* mutation carriers, though significantly lower than those recorded in *LMNA* mutation-negative subjects (Table 1).

**Fig 3:**
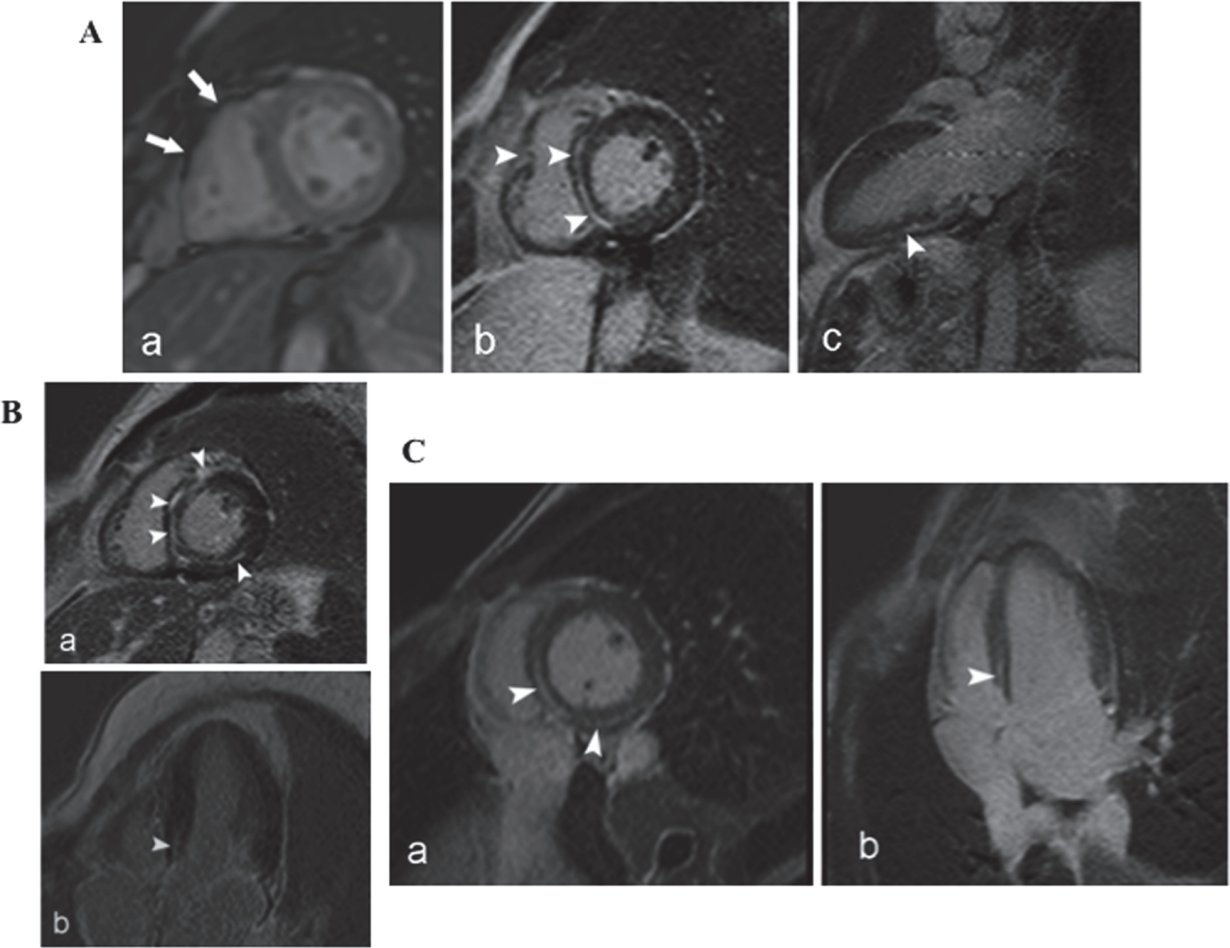
CMR imaging of *LMNA* mutation-positive family members. (A) Short-axis cine (a) and LGE sequences in the Short-axis (b) and 2-chambers long-axis views (c). Bulging (arrow in a) and LGE of the RV free wall (arrowheads in b). Linear midwall LGE is localized at the interventricular septum and LV inferior wall (arrowheads in c) (subject III-5). (B) and (C) LGE sequences in the short axis (a) and 4-chambers long axis views (b). LGE with linear midwall pattern is shown on the LV inferior wall and basal interventricular septum (arrowheads) (subjects III-8, and IV-2).

CMR imaging was performed in 15 subjects, 7 of whom carried the mutated *LMNA* variant. Four of these 7 (57%) had RV involvement with a reduced RVEF (Table 2), and one (subject III-5) had dyskinetic areas with bulging at the RV free wall (Fig. 3Aa; for video file, see S1 Movie). Taken together, mean RVEF values in *LMNA* mutation carriers were significantly reduced in comparison with those assessed in *LMNA* mutation-negative subjects (Table 1). Myocardial fibrosis by late gadolinium enhancement (LGE) imaging was detected in 4 of 7 (57%) of the *LMNA* mutation-positive patients (Table 2) and none of the 8 *LMNA* mutation-negative subjects (Table 1). LGE-positive and *LMNA* mutation-positive subjects were characterized by older age (39 ± 11 vs. 15 ± 4 years, p = 0.034), and longer PR interval (268 ± 77 vs. 143 ± 6 msec, p = 0.032), compared with LGE-negative and *LMNA* mutation-positive subjects, suggesting an age-related phenotype expression. Furthermore, all LGE-positive subjects had NSVT, and one developed sustained VT. LGE was located in the basal interventricular septum and LV inferior wall, and the pattern was linear and localized in the midwall myocardium (Fig. 3) in all subjects.

Patients receiving ICD, before device implantation, underwent coronary angiography showing normal coronary arteries.

During a median follow-up of 122 (range: 12 to 162) months, five patients (II-7, III-1, III-3, III-5, and IV-2) received an ICD in primary prevention, 4 of whom had AV conduction defects. Subject III-1 underwent ICD implantation, unsuccessful VT transcatheter ablation and, 7 years later, at the age of 45, heart transplantation for both sustained VT recurrences in storm (Fig. 2D) and subsequent LV function deterioration (LVEF 30%). Subject IV-2, at the age of 24 years, received a prophylactic ICD that, 4 months after device implantation, discontinued sustained VT (mean cycle length 281 msec) by antitachycardia pacing. Patient III-8 refused prophylactic ICD implantation and has an implantable loop recorder that detected up to 3.5 sec asymptomatic asystoles (Fig. 2C). During follow-up, the index patient continued to have episodes of NSVT, showed paroxysmal AF documented on ICD memory and, after 13 years from diagnosis, he developed complete AV block (Fig. 2B).

Two of 10 (20%) of the *LMNA* mutation-positive family members (subjects IV-6 and IV-8, respectively aged 14 and 12 years) were asymptomatic, free of significant arrhythmias, and revealed normal cardiac function. All *LMNA* mutation-negative family members were clinically asymptomatic, and phenotype negative after cardiac evaluation (Table 1). We did not observe any overlap with other known laminopathies in this family.

### Characterization of LMNA Mutant in HL-1 Cardiomyocytes

To functionally characterize the newly identified *LMNA* mutation, two constructs were generated to express both WT and mutated LMNA as N-terminally tagged EGFP-fusion proteins. For brevity, the (p.Leu140_Ala146dup) LMNA mutation will be termed “LMNA DUP” in the following results and figures.

To analyze LMNA subcellular localization, as well as the overall organization of the nuclear envelope (NE) upon ectopic LMNA expression, HL-1 cardiomyocytes were transiently transfected with the LMNA constructs then subjected to fluorescent confocal microscopy analysis and co-localization experiments with other nuclear components.

**Fig 4:**
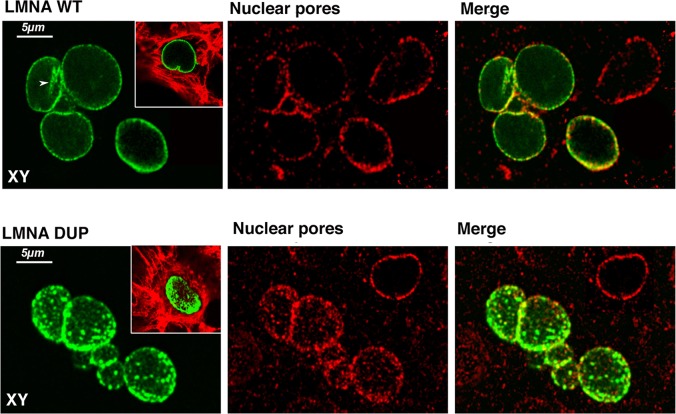
Immunofluorescence confocal analysis of LMNA transfected HL-1 cells. Cells transfected with both LMNA WT and DUP are depicted. LMNA is visualized in green, Nuclear Pores in red, and colocalization in yellow in the merge panels. In the insets, a merged image of LMNA and Phalloidin-TRITC is shown. Planar XY projections were depicted in each experimental condition.

As expected, LMNA WT was uniformly distributed along the NE rim and typically in intranuclear invaginations of nuclear membrane (Fig. 4, LMNA WT, arrow). Cardiomyocytes expressing LMNA WT showed nuclei with regular rounded shapes. Co-localization experiments using antibodies against the nuclear pore complex showed that LMNA WT is tightly associated with nuclear pores (Fig. 4, LMNA WT, Nuclear Pores), which, in turn, showed the regular expected distribution along the whole nuclear periphery.

In contrast, the localization of LMNA DUP appeared profoundly impaired, clearly expressed in aggregates of different sizes, not uniformly distributed along the NE, and notably absent from the intranuclear invagination of the NE. In addition to LMNA disorganization, nuclear pores were also altered in the LMNA DUP-expressing cells, resulting in LMNA organization in clusters (Fig. 4, LMNA DUP, Nuclear Pores).

Interestingly, as assessed by live imaging, both lamin A proteins have the same rate of synthesis and stability in cultured cardiomyocytes. Moreover, western blotting analysis on lysates from the same cells clearly showed that the expression levels of both WT and DUP LMNA constructs were comparable, regardless of the antibody used in the analysis (S1 Fig).

In addition, LMNA DUP-expressing cells were able to normally cycle and divide like LMNA WT-expressing cells (S2 Fig).

### Analysis of Nuclear Envelope Integrity upon Cell Stresses in WT and Mutant LMNA-Expressing HL-1 Cells

In order to assess functional consequences of the altered nuclear lamina structure, the integrity of the NE of LMNA DUP-expressing cells, and its resistance to cellular stress, were checked.

Interestingly, cardiac myocytes, differently than other cell types, do not exhibit the volume regulatory response after exposure to hypertonic conditions [18]. Indeed, an osmotically-induced and uncompensated cell shrinkage may strain the nucleus.

To examine NE integrity in this experimental condition, we monitored the subcellular location of the nuclear marker CellLight Nucleus-Red Fluorescent Protein (NRF, Invitrogen) expressed simultaneously to lamin A proteins in HL-1 cells by transient transfection.

**Fig 5:**
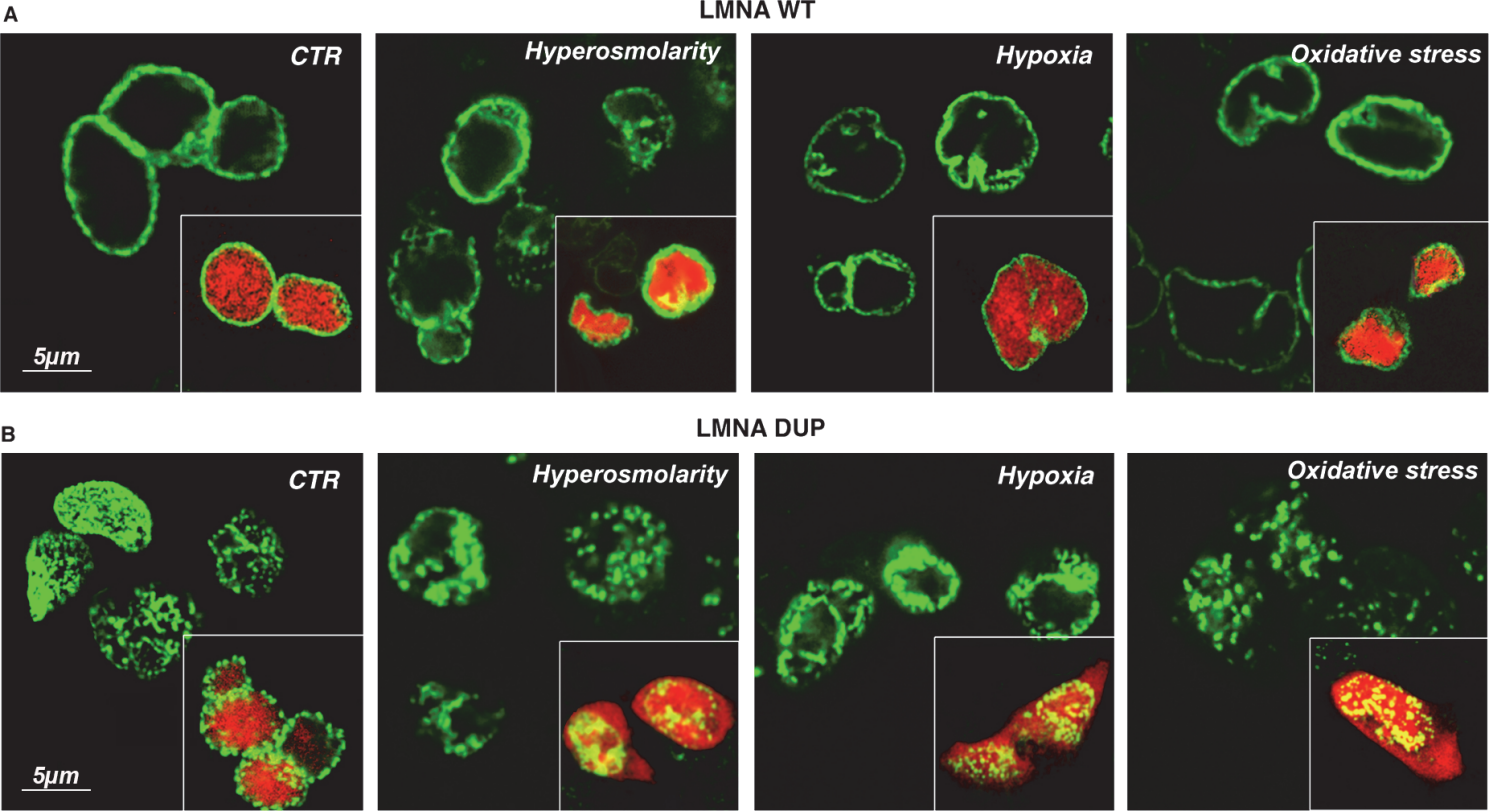
Analysis of nuclear envelope integrity under stressing condition in LMNA transfected HL-1 cells. Nuclear WT LMNA (A) and DUP LMNA (B) signals in control (CTR) and stressing conditions (Hyperosmolarity, Hypoxia, Oxidative stress). The merged signals of LMNA proteins and the RFP nuclear marker are shown in the insets. Confocal XY planar projections are depicted in each experimental condition.

As shown in Fig. 5, the red nuclear marker was confined to the nucleus in both LMNA WT and DUP-expressing cells in control conditions, indicating that nuclear integrity was not significantly impaired in LMNA DUP-expressing cells under resting conditions (Fig. 5, LMNA WT, LMNA DUP, CTR). After 2 h in 300 mM mannitol, added to the culture medium, the nuclear morphology of the LMNA WT-expressing cells, as well as the LMNA WT labelling, was slightly compromised (Fig. 5A, LMNA WT, Hyper), but the nuclear marker was still contained into the nucleus (Fig. 5A, LMNA WT, Hyper, inset); this suggested that NE was not leaky under this challenging condition.

In contrast, in the same condition, extensive nuclear deformations appeared in LMNA DUP-expressing cells and the red nuclear marker escaped from the nucleus into the cytoplasm, suggesting that NE integrity was impaired under this stressing condition (Fig. 5B, LMNA DUP, Hyper, inset).

Interestingly, similar results were obtained when cardiomyocytes were subjected to either hypoxic (5% O2 for 8 h) or H_2_O_2_-induced oxidative stress (300 μM H_2_O_2_ for 4 h), suggesting that the NE of the LMNA DUP-expressing cardiomyocytes was more fragile under different cellular stresses (Fig. 5).

**Fig 6:**
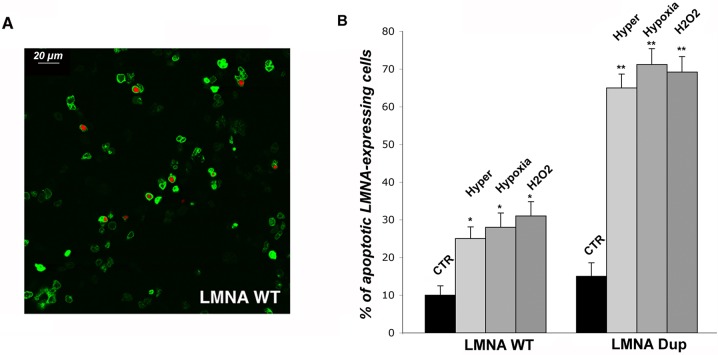
Apoptosis assay in LMNA transfected HL-1 cells. (A) Representative XY confocal planar projections of LMNA transfected cells (green) labelled with EthD-1 (red) in control conditions are depicted. (B) Quantitative analysis of apoptotic cells in control and under hyperosmotic (Hyper), hypoxic (Hypoxia), and oxidative (H_2_O_2_) conditions. Data are reported as % of apoptotic cells (double-labelled cells) in overall LMNA-expressing cells (green labelled cells). Statistical analysis was performed on 3 independent experiments and significance calculated by Student’s T-test for unpaired samples. *P< 0.0002 is relative to CTR vs. stressing conditions in WT LMNA expressing cells and **P < 0.0001 is relative to CTR vs. stressing conditions in DUP LMNA expressing cells.

The expected final consequence of the increased NE fragility under different cellular stressors is apoptosis. We performed the apoptosis assay by using Ethidium homodimer-1 (EthD-1), a membrane-impermeable fluorescent dye, which only enters dying cells with leaky plasma membranes and binds to DNA in the nucleus, emitting red fluorescence (Fig. 6A).

As shown in Fig. 6B, under all the stressing conditions tested, apoptosis increased by about 4 times in cells expressing LMNA DUP, compared to WT LMNA-expressing cells.

We then analysed whether the canonical Wnt/β-catenin signalling was altered in cells expressing LMNA DUP to tentatively identify the pathomechanism underlying this form of cardiomyopathy.

When we analysed **β**-catenin localization and phosphorylation levels in LMNA-expressing HL-1 cells, we found that β-catenin was localized to the cell-to-cell contacts in HL-1 cardiomyocytes expressing either WT or mutated lamin A (S3A Fig). Moreover, the amount of phospho-β-Catenin was unchanged upon LMNA DUP expression in HL-1 cells even under hypoxic conditions, suggesting that the canonical Wnt signaling pathway was not suppressed in LMNA DUP-expressing cardiomyocytes (S3B Fig).

## Discussion

In this study, we identified a novel lamin A/C gene mutation associated with a familial form of arrhythmogenic cardiac laminopathy and characterized it, defining a possible pathogenic mechanism leading to disease development.

Mutations in the *LMNA* gene account for approximately 6–8% of all DCMs and 33% of DCM cases in association with cardiac conduction defects [4, 19–21]. In recent years, mutations of the lamin A/C gene associated with an ARVC-related phenotype were found [8, 22]. Moreover, a combination of morphofunctional phenotypes between DCM and ARVC were highlighted, suggesting a new classification of cardiomyopathies [23].

The newly identified mutated *LMNA* variant can be convincingly considered causative of the clinical features observed in this family for several reasons. First, *LMNA* is a disease gene for both cardiac laminopathies and ARVC [4, 8, 22]. Additionally, a co-segregation of the novel lamin A/C mutation with the disease phenotype was observed within the family. The subjects carrying the *LMNA* variant displayed arrhythmogenic cardiomyopathy of different phenotypes, including ARVC, DCM, LV systolic dysfunction without LV enlargement, system conduction defects, and arrhythmias, showing intra-familial variability of the cardiac phenotype [4, 13]. Importantly, we documented NSVT in 60%, and conduction system disturbances in 70%, of *LMNA* mutation-positive family members, emphasizing the value of family genetic screening to identify silent mutation carriers [13] and the need for tailored clinical monitoring aimed to undertake early treatment strategies and prevent sudden death [24]. In agreement with previous studies [4, 13, 20], two *LMNA* mutation-positive family members under the age of 20 years have no evidence of cardiac structural abnormalities, thus suggesting incomplete and age-related penetrance of the mutation. Moreover, absence of the mutation was associated with normal clinical status in all evaluated relatives of the index patient.

The newly detected *LMNA* variant is in an amino acid region localized to coil 1B of the central α-helical rod domain of the lamin A/C proteins, highly conserved among several species, and was not found in 500 control chromosomes or in the aforementioned databases of genetic variants (see Methods).

In our study, the genetic and clinical data for *LMNA* mutation in this family were strengthened with functional studies. The *in vitro* characterization of this new *LMNA* variant showed that mutated LMNA loses the uniform expression along the nuclear rime and perturbs nuclear shape and nuclear pore complex organization in cultured cardiomyocytes in resting conditions. The loss of the higher-order assembly of lamin mutated polymers probably leads to a loss of nuclear stability and enhanced sensitivity to mechanical strain [25, 26]; this LMNA mutant significantly increases nuclear envelope fragility upon different cellular stresses, such as hypertonic, hypoxic, and oxidative stresses. The leakage of NE in mutated lamin A-expressing cardiomyocytes under hypertonic conditions suggested a decreased mechanic resistance of the NE. A similar nuclear fragility was observed under hypoxic and oxidative stresses. It is indeed possible that this newly identified LMNA mutation drastically decreases both the tolerance and the adaptation of myocardium to stressing conditions, making cardiomyocytes more susceptible to nuclear breakage and cell death during mechanical stress [26].

It is recognized that lamin A is involved into physical and functional connections between the nucleus and cytoskeleton required for effective mechanotransduction in cells (for review, see [2]). It is indeed possible that the mutated lamin A causes not only a decreased mechanic resistance of the NE but also altered nuclear-cytoskeletal coupling with impairment of the mechanotransduction machinery. In hypoxic conditions, in which beating frequency and cellular work increase in cardiomyocytes [27], continuously underwent to mechanical strain due to contraction cycles, the impairment of the nuclear- cytoskeletal connection may result in inappropriate constraints onto the NE, which can, in turn, lose its integrity due to expression of the mutated lamin A. Moreover, it was reported that the nuclear-cytoplasmic compartmentalization can be profoundly affected by ROS, including H_2_O_2_, since nuclear transport factors are the primary cellular targets for oxidants [28]. This effect, together with the nuclear pore clustering induced by the expression of mutated lamin A, may further affect the selective permeability of the NE, ultimately inducing massive nucleoplasm leakage, as observed in our functional studies.

Importantly, the impairment of the nuclear-cytoskeletal connection, due to the expression of the mutated lamin A, may increase the energy cost of contraction and oxygen demand, thus mimicking hypoxic stress even in the absence of physical exercise. Moreover, it has been reported that, in cardiomyocytes, hypoxic conditions increase ROS species production [29], suggesting that both hypoxic and oxidative stresses in mutated lamin A-expressing cardiomyocytes can be continuously induced, even in resting conditions.

In addition to the decrease in mechanic resistance to stressing conditions, it is possible that the newly-identified LMNA makes cardiomyocytes more prone to pro-apoptotic pathways, speeding up the cardiomyocyte apoptotic process once initiated by a stressing condition.

One of the intracellular pathways altered in forms of ARVC due to desmoplakin mutations is the canonical Wnt/β-catenin signalling [30]. Suppression of this pathway induces adipogenesis, fibrogenesis, and apoptosis, the histological hallmark of the disease [31, 32].

However, we found that the canonical Wnt signaling pathway was not suppressed in LMNA DUP-expressing cardiomyocytes. Further experiments will be necessary to identify the intracellular pathway/s involved in the pathogenesis of the cardiolaminopathy described in this study.

Regardless of the pathways, we showed that the final fatal consequence of this *LMNA* mutation is cell death under cell-stressing conditions.

Cardiomyocyte apoptosis may lead to the development of arrhythmias, potentially resulting in sudden cardiac death [33]. An arrhythmogenic effect of apoptosis may be mediated in at least two ways. First, in the progress of dying, a cardiomyocyte passes through phases of increased excitability or becomes automatic, at least until it is dead. Second, from a random grouping of several such dead cardiomyocytes, the process of normal activation in that area of heart muscle must be deranged and redirected in a way that would provide a suitable anatomical substrate for re-entrant arrhythmias (for review, see [34]). Sudden cardiac death in patients with *LMNA* mutations may occur due to ventricular arrhythmias, bradyarrhythmias, or asystole [4, 35]. Previous studies suggested that apoptosis in system conduction cardiomyocytes could cause either tachyarrhythmias or bradyarrhythmias, including complete AV block, as observed in our patients, probably playing an important role in the pathogenesis of sudden cardiac death [33].

Pathophysiological mechanisms leading to the cardiac phenotypes caused by *LMNA* mutations are not yet fully understood [25, 26]. Our experimental data shed light on the clinical findings we collected. In this study, the majority of *LMNA* mutation-positive subjects had ventricular arrhythmias and/or conduction system defects, including severe arrhythmic phenotypes, such as sustained VT and complete AV block, while cardiac function was variable. These findings are in line with previous observations showing that cardiac laminopathies carry high arrhythmogenic risk, even if left ventricular ejection fraction is preserved [4, 24, 35–38].

In our study, myocardial fibrosis by LGE-CMR was found in four *LMNA* mutation carriers who had documented NSVT, one of whom developed sustained VT, and 3 who showed conduction system disturbances. These findings agree with a recent study that included 41 lamin A/C mutation-positive subjects and showed association of myocardial septal fibrosis with ventricular arrhythmias and a prolonged PR-interval [39]. Furthermore, the typical pattern of LGE detected by CMR imaging in our patients was linear and midwall, predominantly located in the basal interventricular septum, consistent with the distribution of myocardial fibrosis previously described in lamin A/C mutation-positive patients [39, 40].

Taken together, our clinical, genetic, and functional data allow us to hypothesize a possible disease mechanism by which the mutated *LMNA* variant causes decreased nuclear stability and impaired nuclear-cytoskeletal coupling, resulting in a higher susceptibility/sensitivity to nuclear rupture and cardiomyocyte apoptosis in tissue subjected to mechanical stress, like the heart [21, 31, 32, 36, 41]. Apoptosis may lead to heterogeneity of cardiac conduction and dispersion of refractoriness [42], providing a basis for the arrhythmias we observed in our patients. In addition, throughout the disease course, cardiomyocyte loss, likely repaired by replacement fibrosis, may provide a possible substrate for conduction block and re-entrant arrhythmias [42, 43]; this hypothesis is in line with previous studies showing that *LMNA* mutation carriers with conduction defects and arrhythmias have myocardial fibrosis involving cardiac conduction system, documented by histopathological examinations [21, 36, 41].

Our study suggests that myocardial fibrosis detected by LGE-CMR may be considered a marker of higher arrhythmic risk in patients with *LMNA* mutations, contributing to identify those that would benefit from ICD implantation, in agreement with recent clinical findings [39, 44].

However, there is growing evidence that life-threatening arrhythmias or sudden cardiac death may occur without myocardial fibrosis in arrhythmogenic cardiomyopathy [42, 45]. In these cases, apoptosis-related enhanced excitability may play a role, which must be proved in further studies.

Some limitations of this study need to be mentioned. The findings of myocardial fibrosis by CMR imaging could be only documented in our patients without pacemaker or ICD. Moreover, LGE-negative and *LMNA* mutation-positive family members were significantly younger than LGE-positive subjects, suggesting an age-related cardiac phenotype expression. Future larger studies, including CMR evaluations and long-term follow-up of healthy mutated subjects, should help to elucidate the timing of expression of the phenotypic traits.

## Conclusions

In conclusions, our functional data, combined with clinical and genetic findings, indicate *LMNA* p.Leu140_Ala146dup as a disease-causing mutation, and suggest cardiomyocyte apoptosis as a possible molecular mechanism leading to the clinical features observed in this family, thus confirming the emergent role of the *LMNA* gene in the pathogenesis of a wide spectrum of cardiac laminopathies. The current family is a striking example of the possibility of shared cardiac phenotypes between laminopathies and arrhythmogenic cardiomyopathy. The major clinical implication of our findings is that the *LMNA* gene should be included in mutational screening of patients with suspected arrhythmogenic cardiomyopathy, particularly when they have ECG evidence for conduction defects and/or myocardial septal fibrosis on CMR. These results, by integrating clinical, genetic, and functional data, could contribute to future studies aimed at improving risk stratification algorithms and testing possible tailored therapeutic approaches in patients with *LMNA* mutations.

## Supporting Information

S1 Fig LMNA protein biosynthesis dynamics. (A) To verify whether the mutated LMNA shows impaired biosynthetic pathways compared to the wild-type (WT) lamin protein, HL-1 cells were transiently transfected with plasmids containing the WT or alternatively the mutated variant of lamin A and then monitored by live imaging for 16 h in the Biostation device. Both proteins appeared within the cells after about 5 h post-transfection and were trackable until the end of the experimental time course, suggesting that both lamin A proteins have, at least in range of time of 16 h, the same rate of synthesis and stability in cultured cells (two representative movie frames were shown). (B) Western blotting analysis on lysates from 24 h transiently transfected HL-1 cells, clearly showed that the expression levels of both WT and DUP LMNA constructs, were comparable regardless of whether the antibody used in the analysis (anti LMNA antibodies, anti EGFP antibodies; anti-GAPDH antibodies; GAPDH was used as loading control).
(TIF)

S2 Fig Cell division visualization. The fate of single nuclei of HL-1 cells transfected with either WT or DUP LMNA construct were depicted as the most representative movie frames at 24, 28, 30 and 32 h of live imaging analysis. The red markers frame LMNA signals to facilitate the nuclei division visualization. The cell was able to normally divide and to survive at the cell division regardless of whether the type of LMNA expressed.
(TIF)

S3 Fig Analysis of β-catenin pathway in either LMNA WT or LMNA DUP-expressing HL-1 cells. A) β-catenin (red) and LMNA constructs (green) co-localization in HL-1 cells. β-catenin was localized to the cell-to-cell contacts in HL-1 cardiomyocytes expressing either WT or mutated lamin A. B) Detection and semi-quantification of phospho β-catenin by western blotting. The amount of phospho β-catenin was unchanged upon LMNA DUP expression in HL-1 cells even under hypoxic conditions, suggesting that the canonical Wnt signaling pathway was not suppressed in LMNA DUP-expressing cardiomyocytes.
(TIF)

S1 Methods This section describes: Cardiac Magnetic Resonance Imaging Protocol, LMNA cloning and vector construction, Cell culture and transient transfection, Western Blotting and Immunofluorescence Confocal analysis, Live imaging analysis, and Stressing assays. (DOC)

S1 Movie CMR imaging movie. The short axis cine sequence of subject III-5 demonstrates bulging of the right ventricle free wall consistent with dyskinesia.
(MOV)
