# Supplementary figures and images for: Clinical and Functional Characterization of a Novel Mutation in Lamin A/C Gene in a Multigenerational Family with Arrhythmogenic Cardiac Laminopathy
Source: PLoS One. 2015 Apr 2;10(4):e0121723. doi: 10.1371/journal.pone.0121723 (PMC4383583; doi:10.1371/journal.pone.0121723)

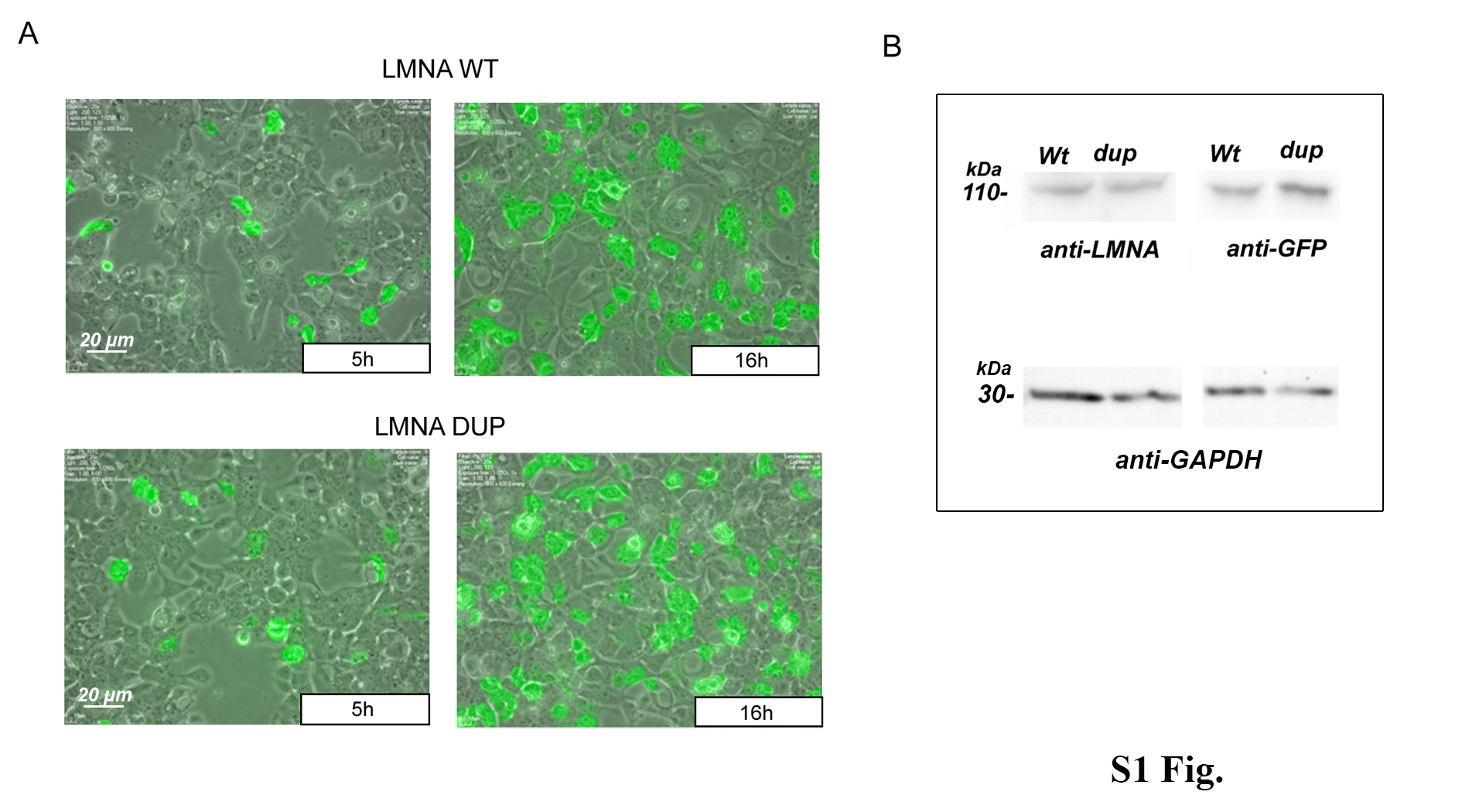

Supplement: S1 Fig — (A) To verify whether the mutated LMNA shows impaired biosynthetic pathways compared to the wild-type (WT) lamin protein, HL-1 cells were transiently transfected with plasmids containing the WT or alternatively the mutated variant of lamin A and then monitored by live imaging for 16 h in the Biostation device. Both proteins appeared within the cells after about 5 h post-transfection and were trackable until the end of the experimental time course, suggesting that both lamin A proteins have, at least in range of time of 16 h, the same rate of synthesis and stability in cultured cells (two representative movie frames were shown). (B) Western blotting analysis on lysates from 24 h transiently transfected HL-1 cells, clearly showed that the expression levels of both WT and DUP LMNA constructs, were comparable regardless of whether the antibody used in the analysis (anti LMNA antibodies, anti EGFP antibodies; anti-GAPDH antibodies; GAPDH was used as loading control). (TIF) [file pone.0121723.s001.tif]

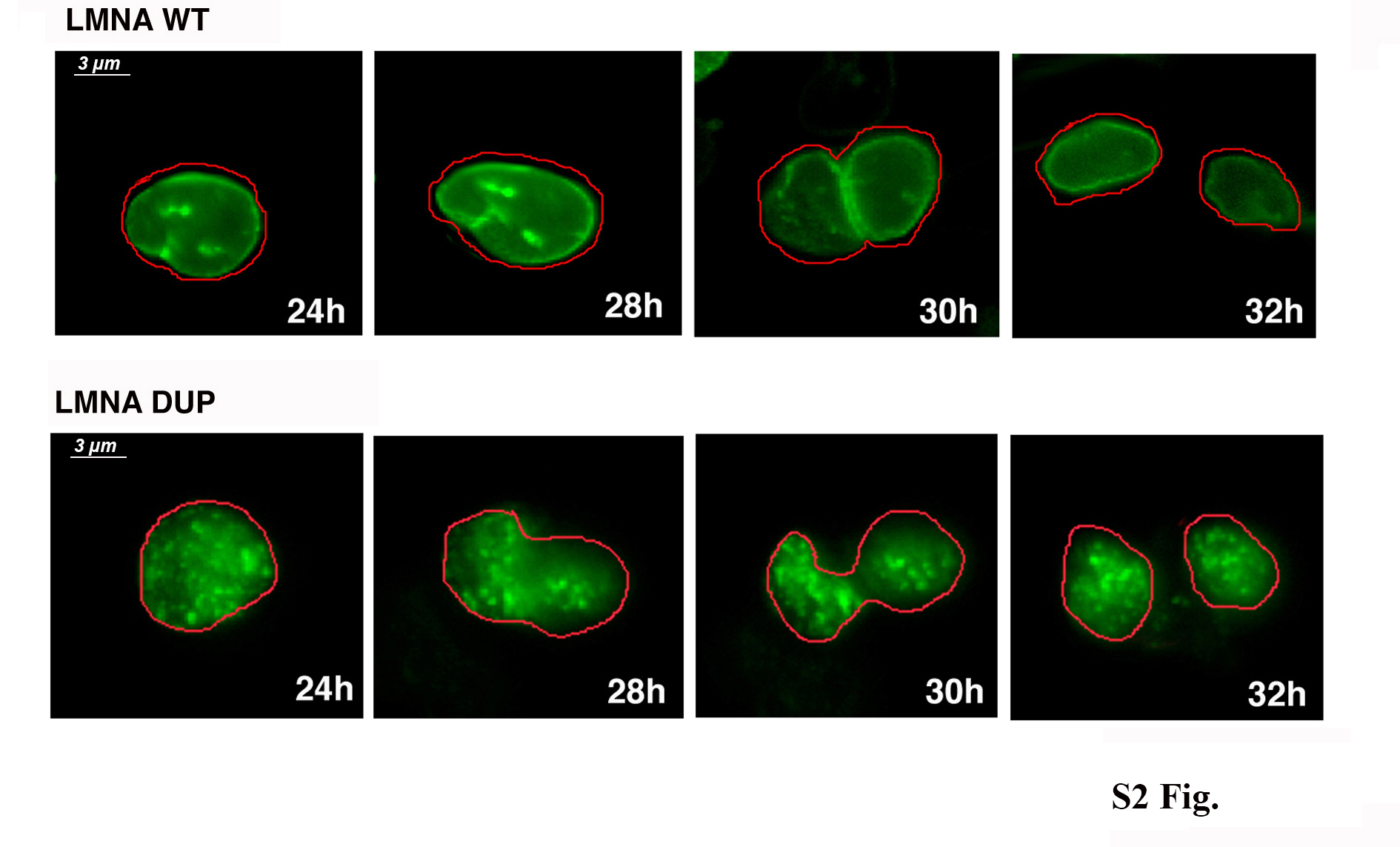

Supplement: S2 Fig — The fate of single nuclei of HL-1 cells transfected with either WT or DUP LMNA construct were depicted as the most representative movie frames at 24, 28, 30 and 32 h of live imaging analysis. The red markers frame LMNA signals to facilitate the nuclei division visualization. The cell was able to normally divide and to survive at the cell division regardless of whether the type of LMNA expressed. (TIF) [file pone.0121723.s002.tif]

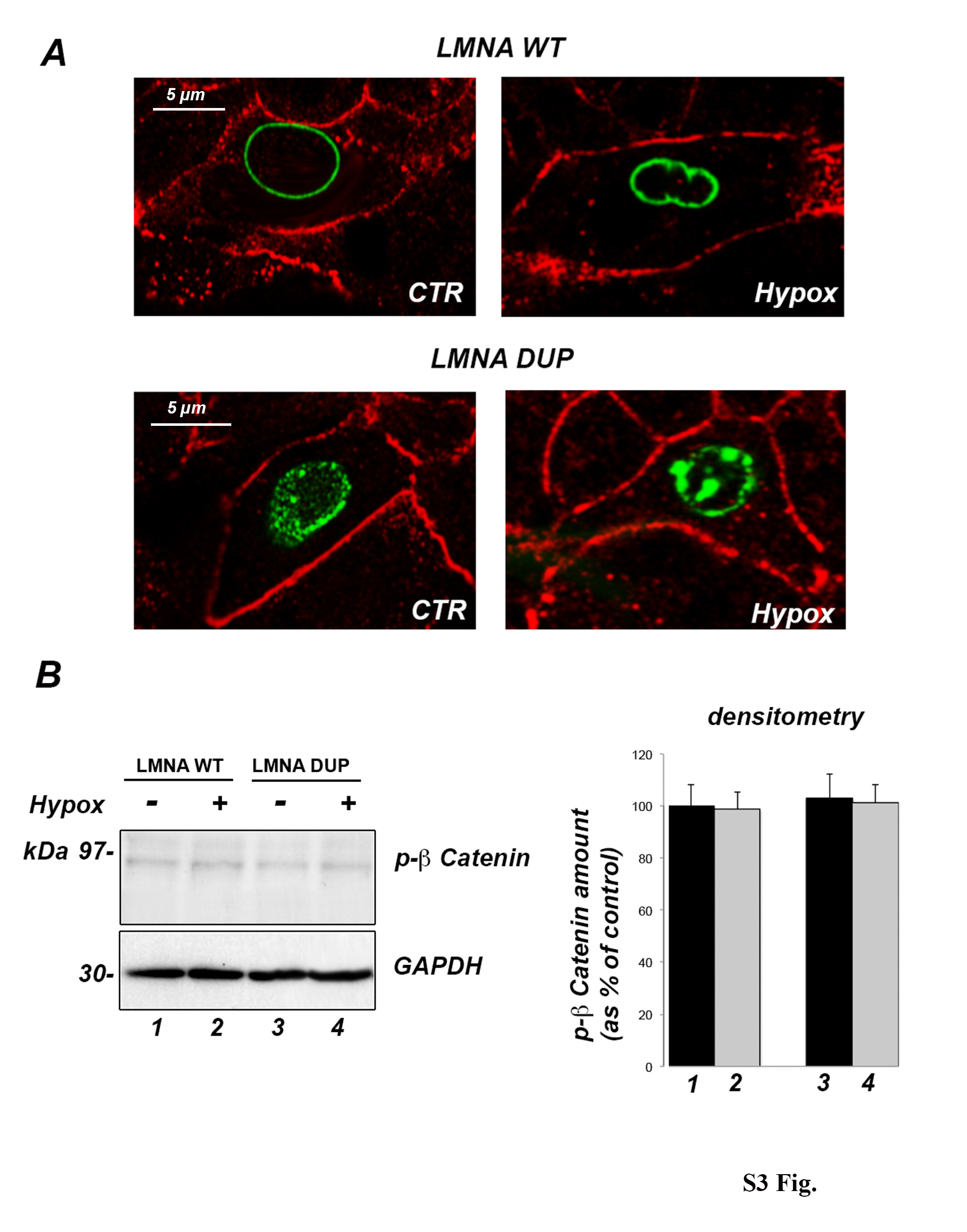

Supplement: S3 Fig — A) β-catenin (red) and LMNA constructs (green) co-localization in HL-1 cells. β-catenin was localized to the cell-to-cell contacts in HL-1 cardiomyocytes expressing either WT or mutated lamin A. B) Detection and semi-quantification of phospho β-catenin by western blotting. The amount of phospho β-catenin was unchanged upon LMNA DUP expression in HL-1 cells even under hypoxic conditions, suggesting that the canonical Wnt signaling pathway was not suppressed in LMNA DUP-expressing cardiomyocytes. (TIF) [file pone.0121723.s003.tif]
