# Supplementary material for: Clinical and Functional Characterization of a Novel Mutation in Lamin A/C Gene in a Multigenerational Family with Arrhythmogenic Cardiac Laminopathy
Source: PLoS One. 2015 Apr 2;10(4):e0121723. doi: 10.1371/journal.pone.0121723 (PMC4383583; doi:10.1371/journal.pone.0121723)
Supplement: S1 Methods — (DOC) [file pone.0121723.s004.doc]

**Supplementary Methods**

**Cardiac Magnetic Resonance Imaging Protocol**

Cardiac magnetic resonance was performed in all the cases on a 1.5-T scanner (Achieva, Philips Healthcare) using an enhanced gradient system (maximum gradient strength 33 mT/m; maximum gradient slew rate, 180 mT ⋅ m−1 ⋅ s^−1^) and a 4-element body phased-array coil. T1 and T2 STIR black blood images as well as cine long-axis four-chamber and two-chamber views were obtained using breath-hold VCG gated sequences. Breath-hold delayed enhanced short-axis and long-axis images were acquired 10 to 15 minutes after an i.v. injection of gadopentetate dimeglumine (Magnevist, Bayer Schering Pharma) (0.2 mmol/kg of body weight) using a turbo field echo inversion- recovery T1-weighted sequence.

**LMNA cloning and vector construction**

RNA was isolated from the peripheral blood of either control or proband as follows. Blood samples (equals roughly 8 ml), were transferred from a BD Vacutainer into a 50 ml polypropylene conical centrifuge tube and the volume brought to 50 ml with Red Blood Cells (RBC) Lysis Buffer (in mM: 155 NH_4_Cl, 12 NaHCO_3_ and 0.1 EDTA). After 15 minutes at room temperature in which RBC went to hypotonic swelling disruption, blood cells (white cells) were pelletted at 600 x g for 10 minutes at 4 °C. The supernatant was carefully removed, the pellet gently resuspended in 1 ml of RBC Lysis Buffer and then transferred to a 1.5 ml microcentrifuge tube. After 2 minutes at room temperature, cells were pelleted for 2 minutes at 3000 rpm. The pellet was subjected to other two cycles of RBC lysis procedure until it resulted completely white. The final cell pellet was washed once in sterile PBS and then resuspended in 800 µl of TRIzol^TM^ solution (Invitrogen). The following steps were performed following the manufacturer's instructions of Trizol ™ reagent.

First-strand cDNA was synthesized, with oligodT primers (Superscript first-strand synthesis system, Invitrogen), from 2 µg of total RNA isolated from the peripheral blood of both control and proband. LMNA full length was amplified using the following primers: forw 5′-GGC AGT CTG CTG AGA GGA AC and rev 5′-GAC ACT GGA GGC AGA AGA GC, which span from exon 5 to exon 11, of LMNA cDNA. The PCR products were gel purified (QIAquick gel extraction kit; QIAGEN) and ligated into the Vivid Colors™ Fluorescent Protein Gateway® Destination Vectors pcDNA™6.2/N-EmGFP-DEST (Invitrogen). This cloning strategy allowed the fusion in frame of the LMNA cDNAs to the next-generation EGFP, Emerald Green Fluorescent Protein (EmGFP) coding sequence at the 5’ end of LMNA coding sequence (N-terminal tail of LMNA protein). Constructs were screened for the correct orientation and fidelity of LMNA sequence amplification by DNA sequencing.

**Cell culture and transient transfection**

Cardiomyocytes HL-1cell line is a generous gift of Prof. William Claycomb (LSU Health New Orleans, USA). They were maintained in culture and seeded for transfection as previously described [1]**.** HL-1 cells were transfected with normal and mutant LMNA constructs using Lipofectamine 2000 reagent according to the manufacturer’s instructions (Life Technologies).

**Western Blotting and Immunofluorescence Confocal analysis**

For gel electrophoresis and immunoblotting, 24 h after transfection cells were extracted in Laemmly Buffer 2X, sonicated for 15 sec and resolved on 10% SDS-PAGE (Novex precast gel; Life Technologies). After protein blotting the membrane was incubated with antibodies against Lamin A/C (Cell Signaling, dil 1:1000), anti GFP antibodies (Covance, dil. 1:500) and anti GAPDH antibodies (Millipore, dil. 1:5000). For the detection of phospho β-catenin in HL-1 cells, 24h after transfection cells were subjected to hypoxia treatment (8h) and simultaneously incubated with Claycomb fresh medium containing the proteasome inhibitor MG132 (25µM) for 5 hours. Cells were then extracted in Antiphosphatase buffer (135mM NaCl, 30mM NaF, 5mM EDTA, 15mM Na_2_HPO_4_, 15mM Na pyrophosphate, 20mM HEPES) containing 1% Triton X-100, Protease Inhibitors cocktails (50X; Roche), 10mM NaF, 1mM Ortho Vanadate, Calyculin A (500 µM) and sonicated for 15 sec. Unbroken cells and nuclei were pelleted by centrifugation at 13,000xg for 30 min at 4°C. Samples, diluted in Laemmli’s buffer, were resolved on 4-12% SDS-PAGE (Novex precast gel; Life Technologies). Western blotting was performed using anti phospho β−catenin (pSer33/pSer37) antibodies (Sigma Aldrich, dil. 1:500) and anti GAPDH antibodies (Millipore, dil. 1:5000).

For immunofluorescence confocal analysis, cells 24h after transfection were fixed in methanol for 6 min at 10 C. After washes with PBS, cells were blocked in saturation buffer (1% bovine serum albumine in PBS) for 20 min at room temperature (RT) and incubated with the antibodies against Nuclear Pore Complex (Covance, dil. 1:500) or antibodies against β-catenin (Santa Cruz Biotechnologies, dil. 1:200) for 2 h at RT in saturation buffer. After 3 washes in PBS cells were incubated with 594 Alexafluor conjugated secondary antibodies (Life Technologies) for 1 h at RT. Confocal images were obtained with a laser scanning fluorescence microscope Leica TSC-SP2.

**Live imaging analysis**

For live imaging experiments cells were seeded in specific Nikon Hi-Q4 glass bottom dishes and experiments were carried out using the BioStation IM device (Nikon), an automatic cell maintenance system, which maintains a stable incubation environment (37°C, 5% CO2, 100% humidity) with scheduled automatic time-lapse image acquisition using phase contrast and two channel epifluorescence optics. Images were acquired with the GFP channel, at 20X or alternatively at 40X of magnification. The acquisition timing was set to every 5 minutes for 16-32 hours and up to 10 cell fields were captured at each time points.

**Stressing assays**

Cells seeded on coverslips were transfected with either WT or mutated LMNA constructs, and at the same time incubated with CellLight^TM^ Nucleus-RFP for the nuclear labeling following manufacturing instruction (Life Technologies). After 24h from transfection and nuclear labeling, cells were subjected to different cell stressing conditions. For hypoxic stress, cells were placed into Hypoxia Modular Incubator Chamber (Billups-Rothenberg Inc) and a flow rate of 4 liters/minute of 100% N_2_ was applied for 15 min [2]. Cells, in the hypoxia chamber saturated with N_2_, were then placed at 37°C for 8h. Cells were subjected to hyperosmotic stress adding Mannitol to culture medium (300 mM final concentration for 2 h) [3].

For oxidative stress, H_2_O_2_ was added to culture medium at final concentration of 300 µM for 4 h [4].

Cells were then rapidly fixed in cold methanol, visualized and analyzed by fluorescent confocal microscopy.

For the apoptosis assay, 24 h after transfection, 1 µM ethidium homodimer-1 (EthD-1) was added at the culturing medium and cells were subjected to the stressing conditions and analyzed as described above. EthD-1 is a membrane-impermeable fluorescent dye, which enters only dying cells with leaky plasma membranes and binds to DNA in the nucleus thus emitting red fluorescence.

The cell death quantization was performed calculating the % of EthD-1 red fluorescent nuclei in LMNA expressing cells by Image J software.

**References**

1. White SM, Constantin PE, Claycomb WC. Cardiac physiology at the cellular level: use of cultured HL-1 cardiomyocytes for studies of cardiac muscle cell structure and function. Am J Physiol Heart Circ Physiol. 2004;286: H823-829.
2. Ngoh GA, Facundo HT, Hamid T, Dillmann W, Zachara NE, [Jones SP](http://www.ncbi.nlm.nih.gov/pubmed/?term=Jones SP%5BAuthor%5D&cauthor=true&cauthor_uid=19023128). Unique hexosaminidase reduces metabolic survival signal and sensitizes cardiac myocytes to hypoxia/reoxygenation injury. Circ Res. 2009;104; 41-49.
3. [Galvez AS](http://www.ncbi.nlm.nih.gov/pubmed/?term=Galvez AS%5BAuthor%5D&cauthor=true&cauthor_uid=12881532), [Ulloa JA](http://www.ncbi.nlm.nih.gov/pubmed/?term=Ulloa JA%5BAuthor%5D&cauthor=true&cauthor_uid=12881532), [Chiong M](http://www.ncbi.nlm.nih.gov/pubmed/?term=Chiong M%5BAuthor%5D&cauthor=true&cauthor_uid=12881532), [Criollo A](http://www.ncbi.nlm.nih.gov/pubmed/?term=Criollo A%5BAuthor%5D&cauthor=true&cauthor_uid=12881532), [Eisner V](http://www.ncbi.nlm.nih.gov/pubmed/?term=Eisner V%5BAuthor%5D&cauthor=true&cauthor_uid=12881532), [Barros LF](http://www.ncbi.nlm.nih.gov/pubmed/?term=Barros LF%5BAuthor%5D&cauthor=true&cauthor_uid=12881532), et al. Aldose reductase induced by hyperosmotic stress mediates cardiomyocyte apoptosis: differential effects of sorbitol and mannitol. J Biol Chem. 2003;278: 38484-38494.
4. Ku HC, Chen WP, Su MJ. DPP4 deficiency exerts protective effect against H_2_O_2_ induced oxidative stress in isolated cardiomyocytes. PLoS One. 2013;8: doi: 10.1371/journal.pone.0054518.
